# Extracellular Vesicle-Associated miRNA in Multiple Sclerosis Subtypes: Differential Profiles in Secondary Progressive Disease and the Effect of One-Year Siponimod Treatment

**DOI:** 10.3390/cells15161441

**Published:** 2026-08-11

**Authors:** Oana Vrînceanu, Smaranda Maier, Doina Manu, Claudia Bănescu, Rodica Bălașa

**Affiliations:** 1 Doctoral School, “George Emil Palade” University of Medicine, Pharmacy, Science, and Technology of Targu Mures, 540142 Targu Mures, Romania; oana.mosora@umfst.ro (O.V.); rodica.balasa@umfst.ro (R.B.); 2 Department of Neurology, “George Emil Palade” University of Medicine, Pharmacy, Science, and Technology of Targu Mures, 540136 Targu Mures, Romania; 3 1st Neurology Clinical, Emergency Clinical County Hospital Targu Mures, 540136 Targu Mures, Romania; 4 Center for Advanced Medical and Pharmaceutical Research, “George Emil Palade” University of Medicine, Pharmacy, Science, and Technology of Targu Mures, 540142 Targu Mures, Romania; doinaromanmanu@gmail.com (D.M.); claudia.banescu@gmail.com (C.B.)

**Keywords:** EV-miRNAs, siponimod, neuroinflammation, secondary progressive MS

## Abstract

Circulating extracellular vesicle-associated microRNAs (EV-miRNAs) are emerging as promising peripheral biomarkers in multiple sclerosis (MS). This prospective, observational pilot study was conceived as a hypothesis-generating investigation to characterize the expression profile of four candidate EV-miRNAs (*miR-223-5p*, *miR-155-5p*, *miR-30a-5p*, and *miR-146a-5p*) within an EV-enriched plasma fraction. The cohort comprised 16 patients with secondary progressive MS (SPMS) undergoing siponimod therapy, 13 age- and sex-matched healthy controls (HCs), and 7 patients with relapsing–remitting MS (RRMS) included as an exploratory comparator. Quantification was performed by quantitative real-time PCR employing the ΔΔC_t methodology, with *miR-16-5p* as the endogenous normalizer. Analyses were conducted cross-sectionally and longitudinally, the latter within a paired subgroup of 11 SPMS patients evaluated at baseline and after twelve months of uninterrupted treatment. Cross-sectional comparisons demonstrated a significant downregulation of EV-miR-223-5p in SPMS patients relative to HCs (fold-change [FC] = 0.26; FDR q = 0.026), whereas EV-miR-155-5p was significantly reduced in both the SPMS (FC = 0.35; FDR q = 0.033) and RRMS (FC = 0.28; FDR q = 0.046) cohorts compared with HCs. No significant intergroup differences were observed for EV-miR-30a-5p or EV-miR-146a-5p. Longitudinal assessment revealed no significant modulation of any target EV-miRNA following one year of siponimod therapy. These preliminary observations should be interpreted with caution, given the exploratory nature and modest cohort size. Importantly, the isolation of total plasma EVs does not permit resolution of the specific cellular provenance of the observed signals, nor does it capture their downstream functional consequences. Nevertheless, the selective downregulation of EV-miR-223-5p and EV-miR-155-5p may tentatively suggest candidate molecular signatures warranting further interrogation. Adequately powered studies incorporating cell-specific EV sorting and paired cerebrospinal fluid sampling will be required to substantiate these signals and clarify their potential utility in monitoring disease progression and therapeutic response in progressive MS.

## 1. Introduction

Multiple sclerosis (MS) is a chronic, inflammatory, and neurodegenerative disease of the central nervous system (CNS) characterized by astrocytosis, microgliosis, and neuronal demyelination. This damage ultimately leads to cell death and causes significant disability in young adults worldwide [1].

MS is a progressive disease characterized by relapses or continuous progression, driven by a cascade of events that triggers a pathogenic immune response [2]. The proinflammatory response in the CNS is believed to be triggered by blood–brain barrier (BBB) disruption. This breakdown of tight junction proteins allows an influx of peripheral immune cells, which drives further inflammation and promotes neurodegeneration [3].

The importance of this compromised communication between the peripheral immune system and CNS cells is underscored by the high efficacy of siponimod, a selective sphingosine-1-phosphate (S1P) receptor modulator [4].

Siponimod (Mayzent) received its initial global approval from the US Food and Drug Administration (FDA) in 2019 for the treatment of relapsing forms of multiple sclerosis, including relapsing–remitting MS (RRMS) and active secondary progressive MS (SPMS). Following its subsequent authorization by the European Medicines Agency (EMA) in January 2020, patient access in Romania was established in April 2022, when the medication was introduced as a fully reimbursed therapeutic option [5].

Pharmacologically, siponimod acts as a selective sphingosine-1-phosphate (S1P) receptor modulator, specifically targeting the S1P1 and S1P5 subtypes. Its mechanism of action is twofold. Peripherally, it binds to S1P1 receptors on lymphocytes, preventing their egress from lymphoid tissues and thereby reducing the infiltration of autoreactive immune cells into the CNS. Centrally, its lipophilic nature allows it to cross the BBB, where it binds to S1P5 and S1P1 receptors expressed on oligodendrocytes, astrocytes, and microglia, exerting direct neuroprotective and potentially remyelinating effects [4].

Extracellular vesicles (EVs) are ubiquitously produced and endogenously secreted membrane-bound particles released by all known cell types [6]. Capable of systemic dissemination and crossing the blood–brain barrier (BBB), EVs facilitate critical cell-to-cell signaling and play a vital role in central nervous system (CNS) homeostasis by regulating synaptic activity, myelin restoration, and neuronal repair [7]. To mediate these processes, EVs transport an array of biologically active constituents, including specific proteins, lipids, DNA, and EV-miRNAs [8].

miRNAs are small, non-coding RNA transcripts of approximately 22 nucleotides in length that function as critical post-transcriptional regulators of gene expression. They exert this regulatory control primarily by inducing mRNA degradation or translational repression across both physiological and pathological conditions [9]. Within biological fluids such as serum, plasma, and cerebrospinal fluid (CSF), miRNAs exist in two distinct states: freely circulating (cell-free miRNAs) and encapsulated within EVs (EV-associated microRNAs). The extracellular vesicles encapsulating EV-miRNAs preserve the biomolecular signature of their originating cells, harboring specific lipid and protein profiles that reliably reflect their cellular origin [10,11].

We hypothesize that plasma EV-miRNAs actively drive the pathophysiological progression of MS. The identification of an early, reliable peripheral biomarker indicating inadequate control of neuroinflammatory processes in treated MS patients could significantly optimize therapeutic decision-making amid an expanding landscape of DMTs. The translation of such a biomarker into clinical practice requires a systematic pipeline, progressing from initial discovery and proof-of-concept to the evaluation of clinical outcomes and rigorous validation.

In spite of growing interest in circulating miRNAs as possible biomarkers in MS, data regarding plasma EV-miRNAs in SPMS remain scarce. Moreover, no longitudinal studies have analysed EV-miRNAs in SPMS cases that received siponimod treatment. Thus, our research was designed as an exploratory proof-of-concept study aimed at identifying potential EV-miRNAs associated with SPMS and assessing their temporal stability during one year of treatment.

## 2. Material and Methods

### 2.1. Subject Population

This prospective, observational study was conducted at the First Neurology Clinic of the County Emergency Clinical Hospital of Târgu Mureș, Romania. A total of 29 participants were enrolled in this prospective observational study, comprising 16 patients with SPMS receiving siponimod therapy and 13 age- and sex-matched healthy controls (HCs). A further cohort consisting of seven RRMS patients was integrated into the study for exploratory comparative analysis. We included patients eligible for siponimod treatment who had completed necessary multidisciplinary evaluations (cardiology, dermatology, and ophthalmology). Clinical data and blood samples were collected prior to the first administration and after one year of continuous therapy. Exclusion criteria encompassed the use of systemic corticosteroids within 30 days prior to treatment initiation, the presence of active acute infections, and any history of other autoimmune diseases.

The study protocol was approved by the Ethics Committee for Scientific Research of the “George Emil Palade” University of Medicine, Pharmacy, Science and Technology of Târgu Mureș (Approval No. 2353/26.05.2023) and the Ethics Committee of the County Emergency Clinical Hospital of Târgu Mureș (Approval No. Ad. 7749/05.04.2023). The study was conducted in accordance with the principles of the Declaration of Helsinki. Written informed consent was obtained from all patients or their legally authorized representatives prior to study enrollment.

### 2.2. Blood Collection

Blood sampling was performed by collecting 20 mL of peripheral venous blood into vacuum tubes equipped with a clot activator. These samples were obtained at two predefined intervals: preceding the first dose of siponimod and after one year of therapy. To obtain plasma for subsequent EV isolation, the collected blood samples underwent a two-step centrifugation process within two hours of collection. Initially, the samples were centrifuged at 300× *g* for 10 min at 4 °C. To ensure the complete removal of cellular debris, a secondary centrifugation step was performed at 2000× *g* for 20 min at 4 °C. Following centrifugation, the obtained plasma supernatant was cryopreserved at −80 °C prior to downstream processing.

### 2.3. Isolation and Identification of EVs from Plasma Samples

Isolation and characterization of EVs followed the recommendations set out in the 2023 update of the Minimal Information for Studies of Extracellular Vesicles (MISEV2023) [12]. Plasma-derived EVs were purified using the ExoQuick^®^ ULTRA EV Isolation Kit for Serum and Plasma (Catalog No. EQULTRA-20A-1; System Biosciences, Palo Alto, CA, USA) according to the manufacturer’s instructions, starting from plasma that had first been pre-clarified [13].

Particle morphology and size were assessed by scanning transmission electron microscopy (STEM) on a Hitachi HD-2700 instrument (Hitachi High-Tech Science Corporation, Tokyo, Japan), run at an accelerating voltage of 200 kV with images acquired at 15,000× and 50,000× magnification. Imaging confirmed that the isolated EVs displayed the morphological diversity typically reported for this vesicle population [14].

Additional physicochemical characterization was carried out on a Zetasizer Nano ZS90 (Malvern Instruments, Malvern, Worcestershire, UK). Hydrodynamic diameter and polydispersity index (PDI) were obtained by dynamic light scattering (DLS), while electrophoretic light scattering provided zeta potential values as a measure of surface charge and colloidal stability. Each sample was measured in triplicate, with mean hydrodynamic diameter, PDI, and zeta potential reported per sample.

### 2.4. RNA Extractions and qRT-PCR Analysis

Following the manufacturer’s protocol, EV-miRNAs were isolated using the phenol-based mirVana^TM^ miRNA Isolation Kit (Thermo Fisher Scientific, Waltham, MA, USA) [13]. Subsequently, complementary DNA (cDNA) was generated using the TaqMan^TM^ MicroRNA Reverse Transcription Kit (Thermo Fisher Scientific, Waltham, MA, USA) alongside target-specific TaqMan^TM^ MicroRNA primers [15].

Quantitative real-time PCR (qRT-PCR) was performed on the 7500 Fast Dx Real-Time PCR System (Applied Biosystems, Waltham, MA, USA). Each amplification reaction was assembled using TaqMan^TM^ Fast Advanced Master Mix (Thermo Fisher Scientific, Waltham, MA, USA) in conjunction with individual TaqMan^TM^ MicroRNA Assays targeting *miR-146a*, *miR-30a*, *miR-155*, and *miR-223*, each comprising sequence-specific primers and a TaqMan hydrolysis probe. Expression levels were normalized to the endogenous small RNA *miR-16-5p*, which served as the internal reference control.

Expression levels were quantified using the delta cycle threshold (ΔCq) method, whereby the cycle threshold (Cq), defined as the number of amplification cycles required to generate a detectable fluorescent signal above background, was determined for each target. Normalized expression values were subsequently derived by subtracting the Cq of the endogenous reference gene *miR-16-5p* from the Cq of each target microRNA, yielding the corresponding delta cycle quantification (ΔCq) values.

### 2.5. Statistical Analysis

All statistical analyses were performed in Python (version 3.14.5) using the scipy, pandas, pingouin, and matplotlib libraries. Group differences were assessed using Kruskal–Wallis and Mann–Whitney U tests, with Welch *t*-tests applied as a parametric complement. Effect sizes were estimated using Hedges’ *g*. Inter-target correlations were evaluated using Spearman rank correlation coefficients. Multiple testing correction was performed using the Benjamini–Hochberg false discovery rate (FDR) method, with statistical significance defined as q < 0.05.

## 3. Results

Expression of the four target miRNAs was normalized to the endogenous control *miR-16-5p* using the ΔCq method. Prior to differential expression analysis, the stability of the reference gene was assessed across cohorts. Expression was normalized to the endogenous reference gene *miR-16-5p* using the double-delta cycle threshold (ΔΔCt) method. The formula applied was: ΔΔCt = ΔCt [Ct(miRNA) − Ct(*miR-16-5p*)] patient − ΔCt [Ct(miRNA) − Ct(*miR-16-5p*)] control. Relative expression (fold-change) was subsequently calculated as 2^−ΔΔCt^. Values below 1 indicate downregulation of the target miRNA in the patient group relative to healthy controls, while values above 1 indicate upregulation.

Reference gene stability was assessed across cohorts prior to differential expression analysis. A three-group omnibus Kruskal–Wallis test showed a significant difference in *miR-16-5p* Ct values across cohorts (H = 11.55, *p* = 0.003), driven by lower Ct values in the RRMS subgroup (mean 27.28 ± 2.00) compared to HC (30.08 ± 1.27) and SPMS (29.86 ± 1.31). Importantly, when HCs were compared against all MS patients combined (SPMS + RRMS), the reference gene showed no significant difference (Mann–Whitney U = 198.5, *p* = 0.110; Welch *t*-test *p* = 0.068), confirming adequate normalization stability for the primary disease-state comparison.

**Figure 1:**
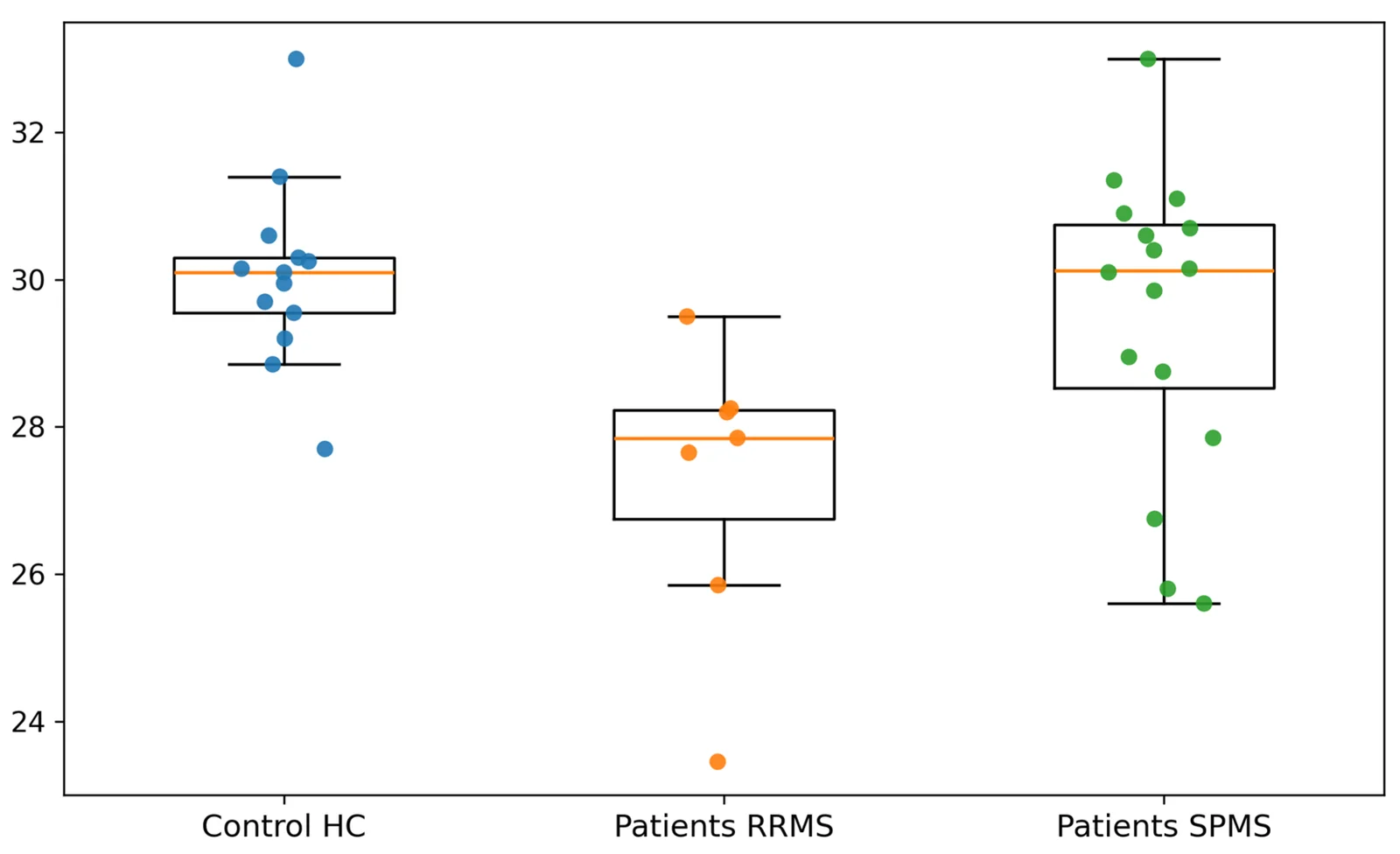
Reference gene (*miR-16-5p*) quality control: Ct distribution by cohort. RRMS patients (orange) show notably lower Ct values (higher abundance) compared to HC (blue) and SPMS/Numeric (green), confirming the group-specific reference gene instability. HC and SPMS distributions were broadly comparable.

Following *miR-16-5p* normalization, group-level descriptive statistics, ΔCt distributions, and fold-changes relative to HC were obtained and are summarized in Table 1 and Figure 1. Higher ΔCt values indicate lower relative expression. *miR-223-5p* and *miR-155-5p* showed the most pronounced elevation of ΔCt values in MS patients relative to HCs, reflecting consistent downregulation. *miR-223-5p* was markedly downregulated in both SPMS (fold-change = 0.26) and RRMS (0.54), while *miR-155-5p* was reduced in SPMS (0.35) and RRMS (0.28). *miR-30a-5p* showed a moderate reduction only in RRMS (0.48), and *miR-146a* expression was essentially unchanged in RRMS relative to HC (fold-change = 1.01) with a modest reduction in SPMS (0.68).

**Figure 2:**
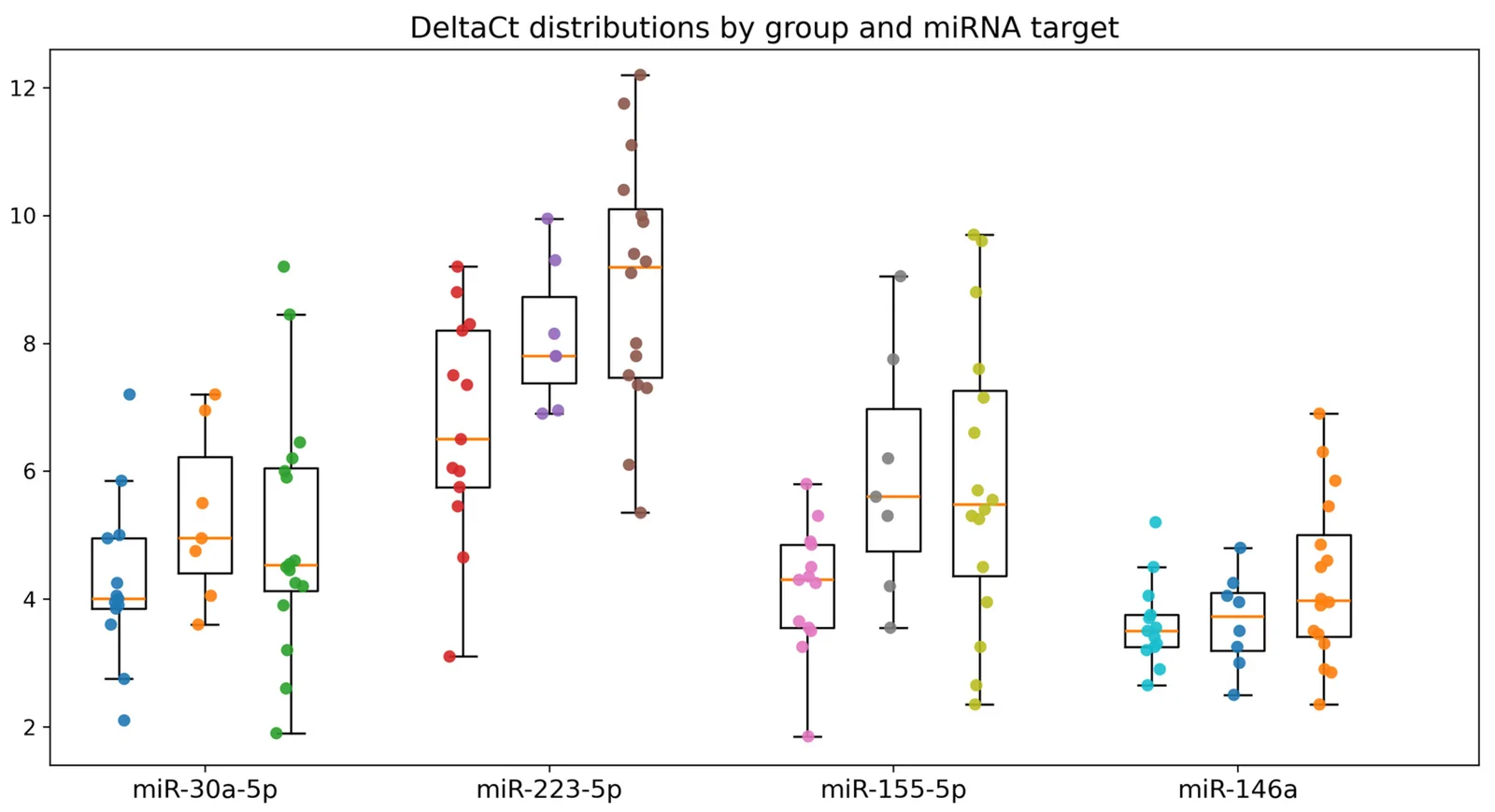
ΔCt distributions for all four miRNA targets across cohorts (control HC, RRMS, SPMS left to right within each target group). Lower ΔCt indicates higher relative expression. *miR-223-5p* and *miR-155-5p* show a progressive increase in ΔCt from HC through RRMS to SPMS, consistent with downregulation in MS patients.

Three-cohort Kruskal–Wallis omnibus testing of subject-level ΔCt values with Benjamini–Hochberg FDR correction (Table 2) confirmed that *miR-223-5p* and *miR-155-5p* showed statistically significant multi-group differences (FDR q = 0.024 for both). Neither *miR-30a-5p* (FDR q = 0.177) nor *miR-146a* (FDR q = 0.177) reached significance. These results are illustrated in Figure 2.

Pairwise Mann–Whitney U tests of subject-level ΔCt values against the HC reference group were performed for three patient groupings: RRMS alone (primary pre-specified comparison), SPMS alone, and combined MS patients (SPMS + RRMS). All comparisons were FDR-corrected (Table 3).

**Figure 3:**
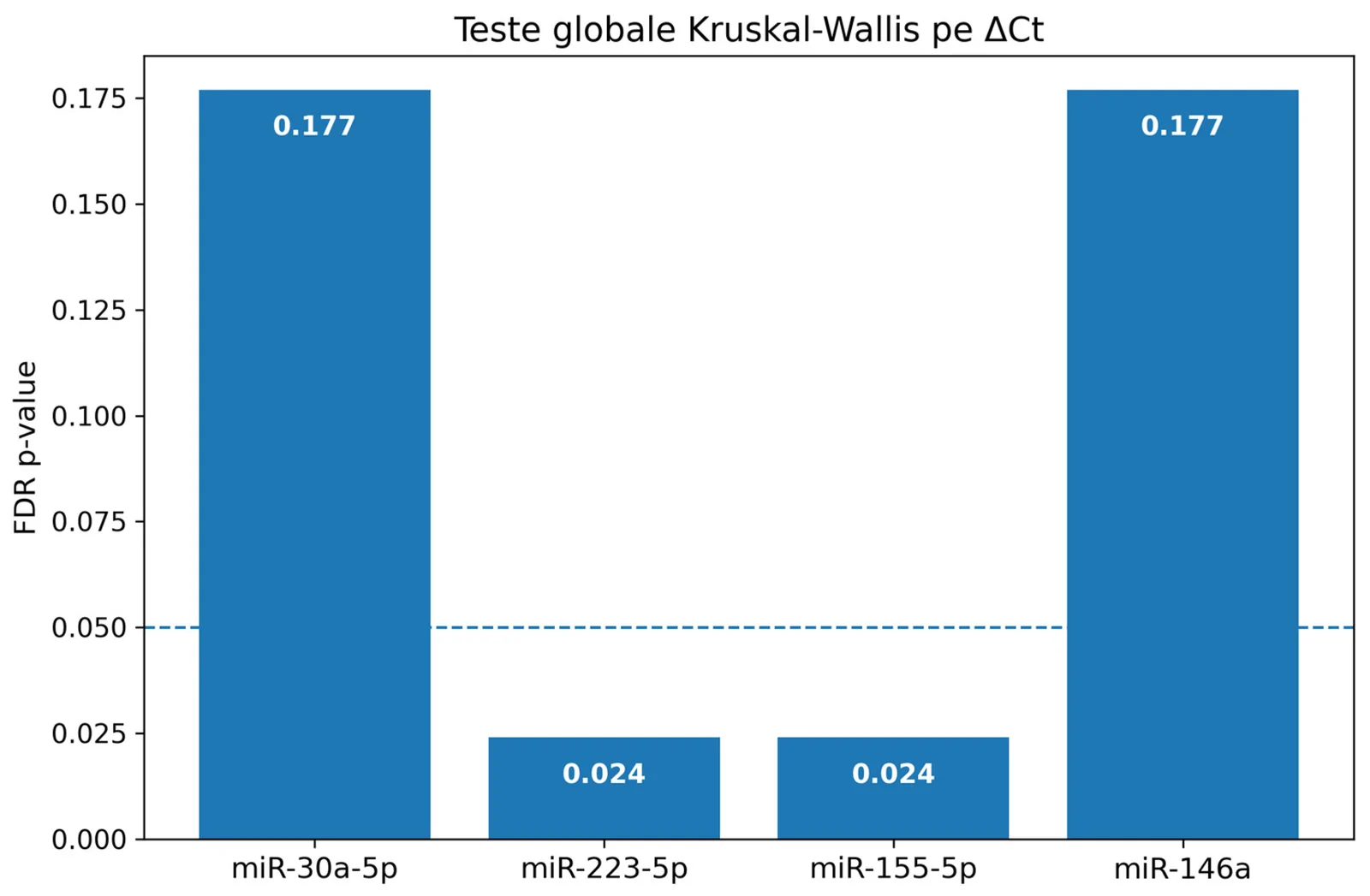
Kruskal–Wallis omnibus FDR-corrected *p*-values for each miRNA target across the three cohorts. The dashed line marks the FDR significance threshold (*p* = 0.05). *miR-223-5p* and *miR-155-5p* (FDR *p* = 0.024 each) exceed this threshold; *miR-30a-5p* and *miR-146a* do not.

*miR-155-5p* was significantly downregulated in all three patient comparisons: SPMS vs. HC (fold-change = 0.32, FDR q = 0.033), RRMS vs. HC (fold-change = 0.28, FDR q = 0.046), and combined patients vs. HC (fold-change = 0.31, FDR q = 0.033). *miR-223-5p* was significantly reduced in SPMS vs. HC (fold-change = 0.26, FDR q = 0.026) and in the combined patient group vs. HC (fold-change = 0.30, FDR q = 0.046), while the RRMS-alone comparison did not reach significance (FDR q = 0.401). Neither *miR-30a-5p* nor *miR-146a* showed significant differences in any comparison (Figure 3).

**Figure 4:**
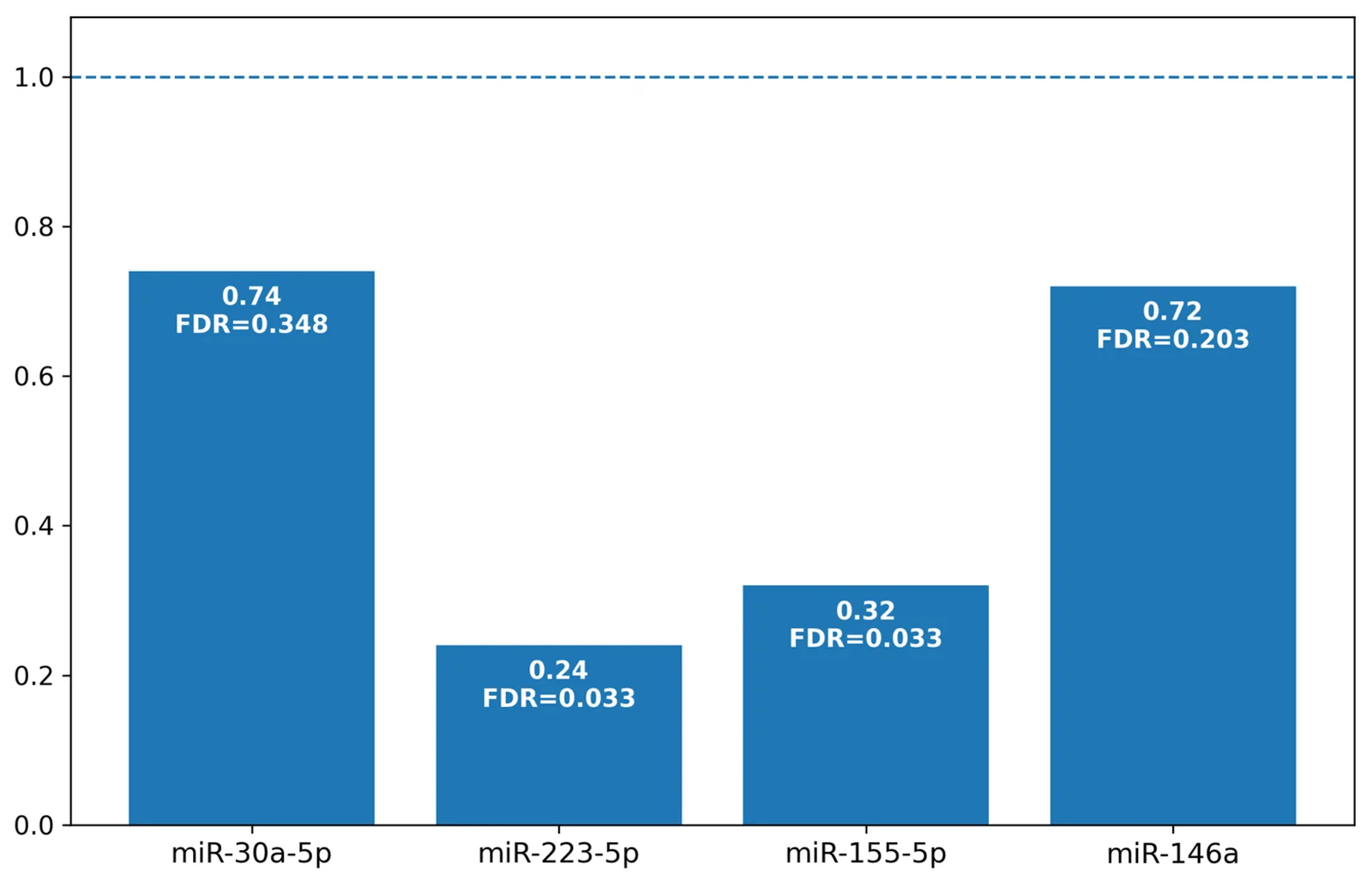
Fold-change (2^−ΔΔCt^) of SPMS patients relative to HC for each miRNA target. *miR-223-5p* (FC = 0.24, FDR q = 0.033) and *miR-155-5p* (FC = 0.32, FDR q = 0.033) are significantly downregulated. *miR-30a-5p* and *miR-146a* do not reach significance.

**Figure 5:**
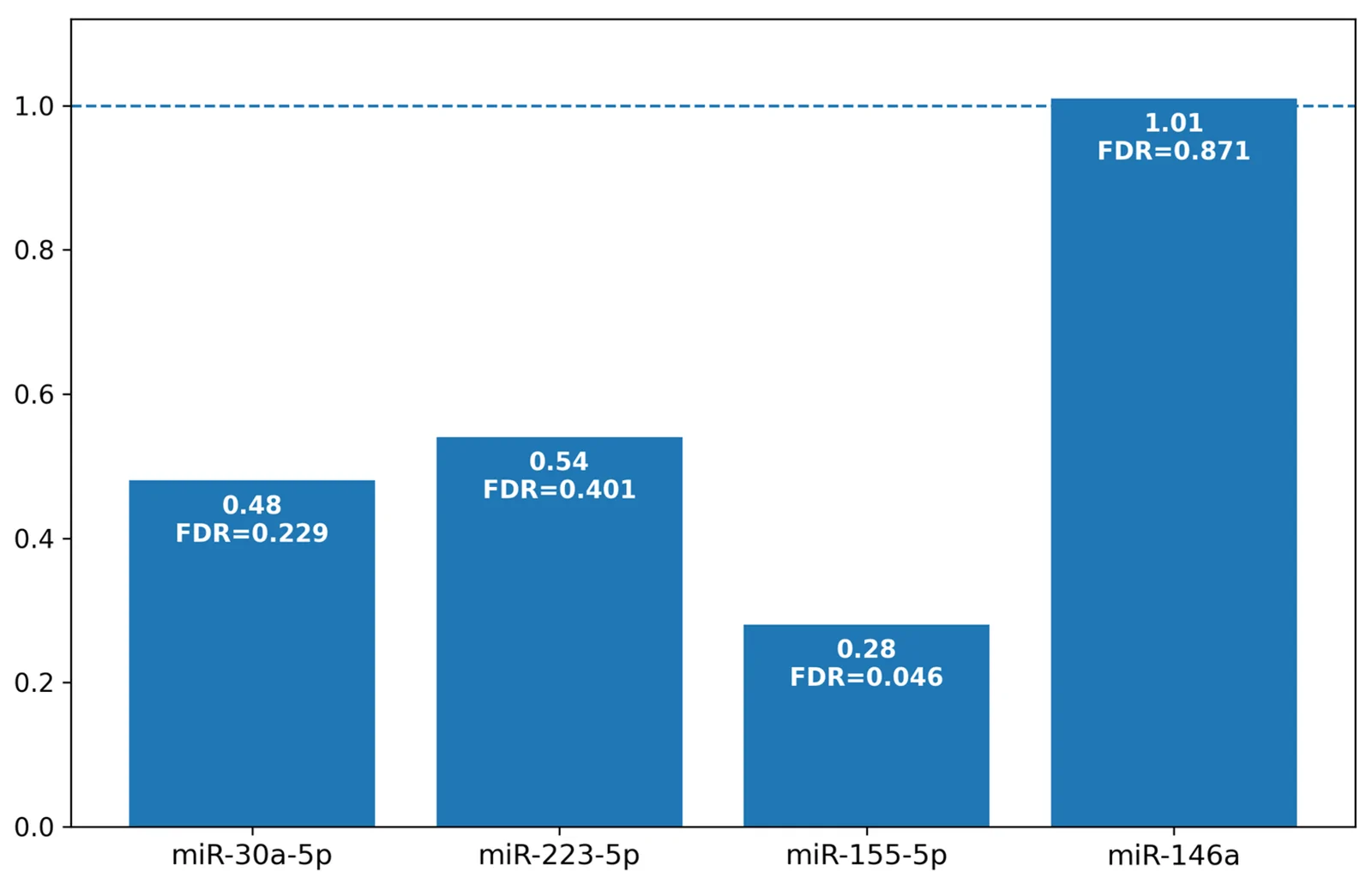
Fold-change of RRMS patients relative to HC. *miR-155-5p* is significantly downregulated (FC = 0.28, FDR q = 0.046). *miR-223-5p* shows a downregulation trend (FC = 0.54) that does not reach significance after FDR correction, likely reflecting the small RRMS group size.

**Figure 6:**
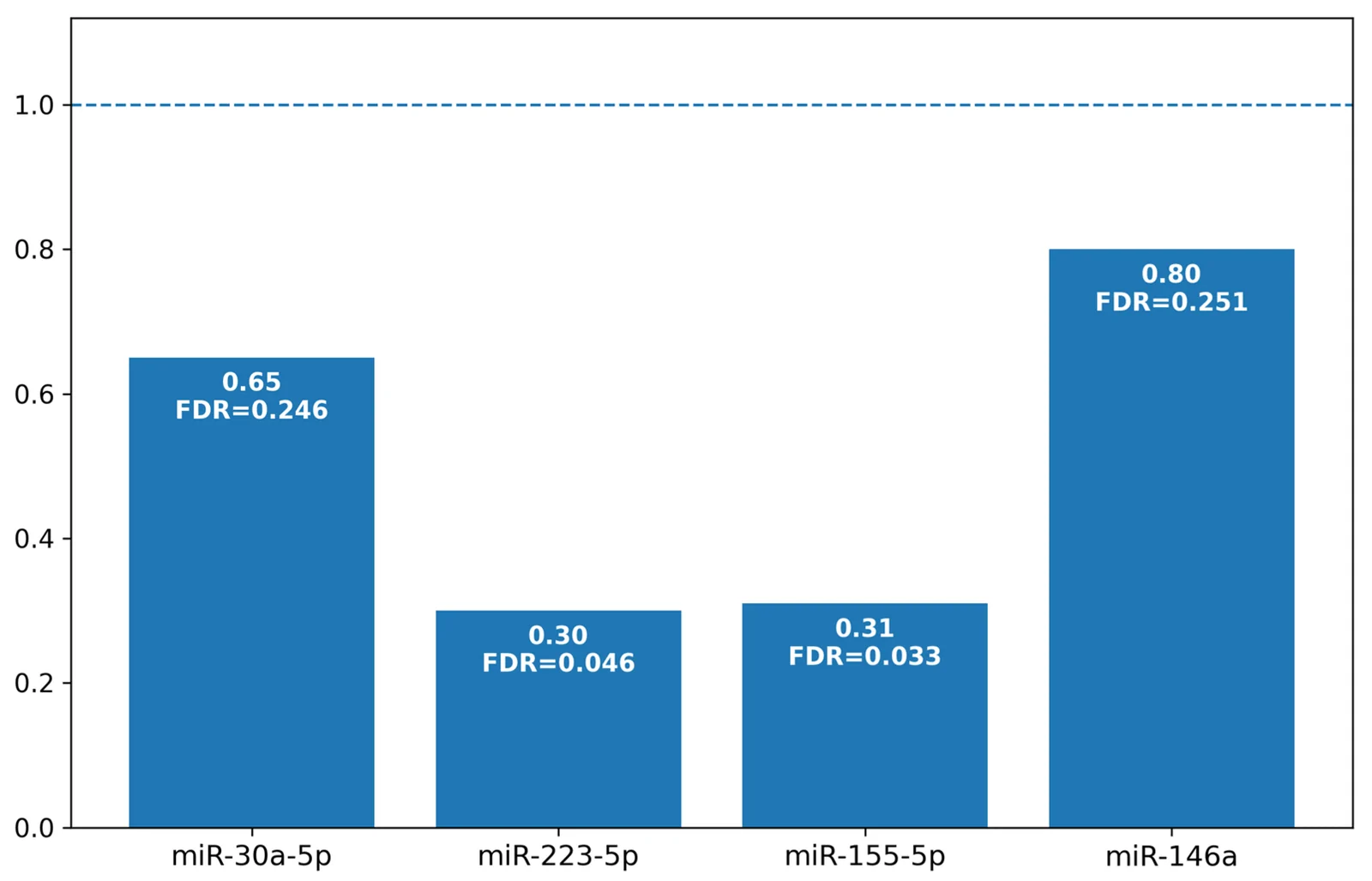
Fold-change of all MS patients combined (SPMS + RRMS) relative to HC. Both *miR-223-5p* (FC = 0.30, FDR q = 0.046) and *miR-155-5p* (FC = 0.31, FDR q = 0.033) reach significance in this combined analysis.

**Figure 7:**
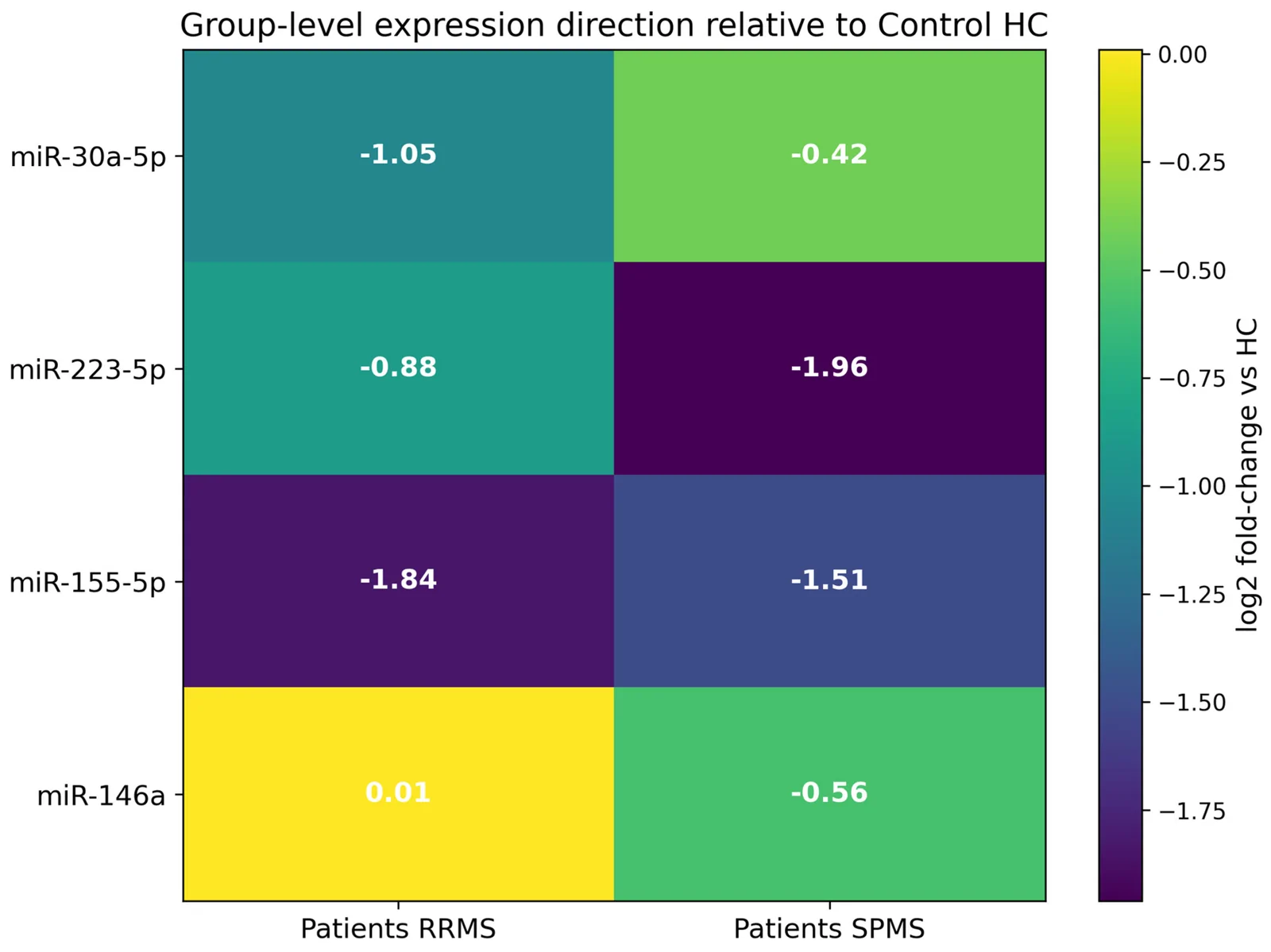
Group-level log_2_ fold-change heatmap relative to HC for RRMS patients and SPMS group. Darker purple indicates greater downregulation. *miR-155-5p* and *miR-223-5p* show the most pronounced downregulation in both patient cohorts, while *miR-146a* is virtually unchanged in RRMS.

The fold-change profiles for the three patient comparisons are shown separately in Figure 4, Figure 5 and Figure 6 and in the combined group-direction heatmap (Figure 7).

**Figure 8:**
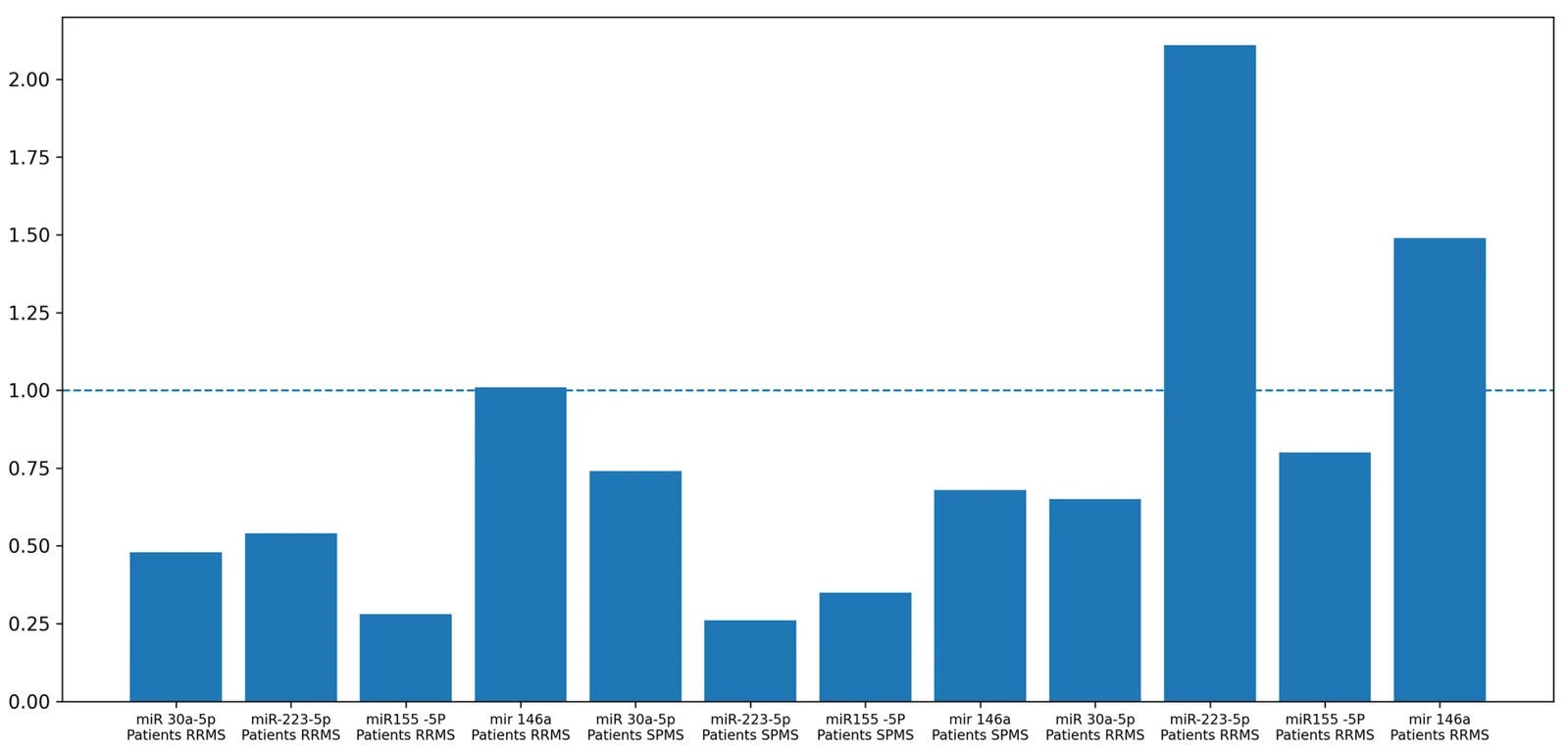
Fold-change overview for all pairwise comparisons across the four miRNA targets. Bars below 1.0 indicate downregulation relative to the comparison group. *miR-223-5p* in the RRMS vs. SPMS direction shows the largest fold-change (>2×), reflecting the difference in severity between the two MS subtypes.

*miR-223-5p* showed the highest ΔCt values and widest distributions across all cohorts, confirming its status as the lowest-abundance target approaching the technical detection limit. The overall fold-change profile across all pairwise comparisons is summarized in Figure 8, which provides a complete overview of the direction and magnitude of differential expression for each target.

To evaluate whether one year of siponimod therapy was associated with changes in EV-miRNA expression, a within-subject longitudinal analysis was conducted in the subset of 11 SPMS patients for whom paired blood samples were available at both baseline (T0, prior to treatment initiation) and after twelve months of continuous therapy (T1). For each target, ΔCq values at T0 and T1 were compared using a Wilcoxon signed-rank test for matched pairs.

**Figure 9:**
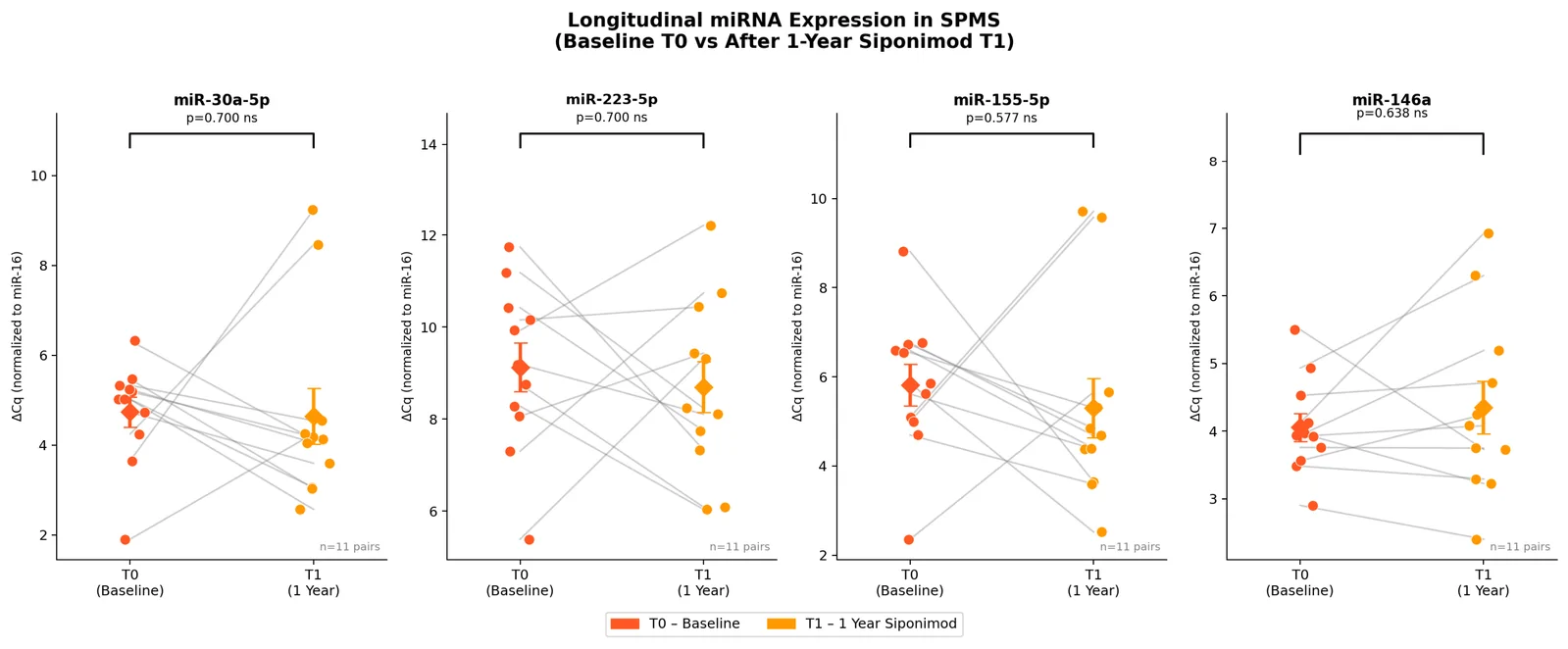
Longitudinal EV-miRNA expression in SPMS patients at baseline (T0, red) and after one year of siponimod therapy (T1, orange). Each line connects the two time-point measurements for an individual patient (n = 11 pairs). Diamonds with error bars represent group means ± 95% CI. The y-axis shows ΔCq normalized to *miR-16-5p*; higher ΔCq values indicate lower relative expression. Wilcoxon signed-rank test *p*-values are annotated above each panel; ns = not significant. None of the four targets showed a statistically significant change after one year of treatment.

No statistically significant change in EV-miRNA expression was detected for any of the four targets over the one-year treatment period (Figure 9). *miR-30a-5p* showed a modest numerical tendency toward lower ΔCq at T1 relative to T0, suggestive of a marginal increase in expression, but this did not reach significance (*p* = 0.700). Similarly, *miR-223-5p* demonstrated a slight reduction in mean ΔCq from T0 to T1 (*p* = 0.700), while *miR-155-5p* showed a comparable non-significant trend (*p* = 0.577). *miR-146a* exhibited the most stable profile across the two time points, with the highest *p*-value of the four targets (*p* = 0.638). In all cases, substantial inter-individual variability was observed, with individual trajectories crossing in both directions and some patients showing increases and others showing decreases in expression between T0 and T1, resulting in wide confidence intervals around the group-level estimates.

## 4. Discussion

This pilot biomarker study investigated the plasma EV-miRNA profiles of *miR-223-5p*, *miR-155-5p*, *miR-30a-5p*, and *miR-146a* in SPMS patients treated with siponimod, comparing them against HCs and an exploratory RRMS cohort, and additionally assessing longitudinal EV-miRNA changes over one year of continuous treatment. The central findings were that both *miR-223-5p* and *miR-155-5p* were significantly reduced in SPMS patients relative to HCs, that *miR-155-5p* was similarly downregulated in RRMS patients, and that neither miRNA showed a statistically significant change after twelve months of siponimod therapy. These results support the concept that plasma EV-miRNAs can reflect the neuroinflammatory milieu of progressive MS and, to our knowledge, provide the first longitudinal EV-miRNA data in siponimod-treated SPMS patients.

The significant downregulation of EV-miR-223-5p in SPMS patients (fold-change = 0.26 relative to HC, FDR q = 0.026) carries important mechanistic implications. *miR-223* is a well-characterized regulator of myeloid cell biology, controlling granulopoiesis, macrophage activation, and the restraint of pro-inflammatory cytokine production through targeting of key transcription factors including *mef-2c* and modulation of the NF-κB pathway [16]. Within the CNS, it has been shown to negatively regulate the *NLRP3* inflammasome in microglial populations, and its experimental deficiency is accompanied by heightened microglial autophagy and worsened demyelination in animal models of MS [17]. The lower EV-miR-223-5p abundance we observed in SPMS may therefore indicate a state of diminished innate immune restraint within the CNS compartment, consistent with the chronic smouldering neuroinflammation that characterises the progressive phase of the disease. This interpretation is supported by prior EV profiling work, which identified *miR-223-3p* as part of a nine-miRNA signature differentiating RRMS from progressive MS subtypes, where it was more abundant in the relapsing phenotype [18]. In a recent review specifically examining EV-miRNAs as transition biomarkers from RRMS to SPMS, *miR-223-5p* was further cited among the miRNAs with the most consistent dysregulation across MS disease stages [19]. The lower EV-enriched fraction abundance of *miR-223-5p* that we observed in SPMS may therefore serve as an indirect, hypothetical indicator of a state of diminished innate immune restraint within the CNS compartment, though this peripheral correlation cannot directly prove or reflect the chronic smouldering neuroinflammation that characterises the progressive phase of the disease. Because our current protocol evaluates total circulating plasma cargo without localizing cellular tissue of origin or capturing downstream functional consequences, this potential link remains an associative hypothesis. Confirming this relationship requires future validation through matching CSF sampling, the enrichment of CNS-specific EV subpopulations, or targeted functional assays.

**Figure 10:**
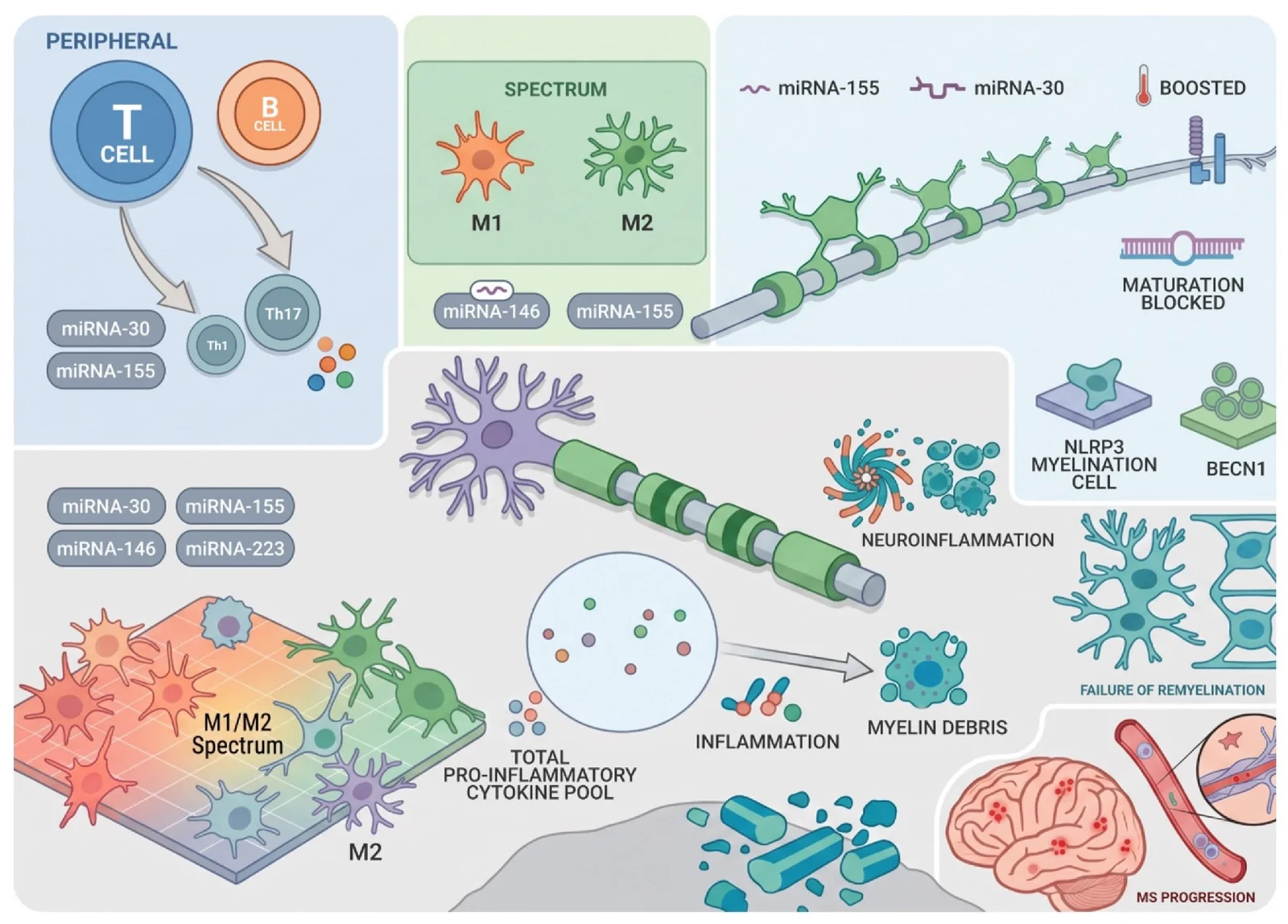
The four miRNAs investigated in the present study participate in interconnected inflammatory and neurodegenerative pathways in MS. In the peripheral compartment, *miR-155* and *miR-30* promote Th1/Th17 T cell polarization, while centrally, *miR-146a* and *miR-155* regulate microglial M1/M2 polarization and oligodendrocyte maturation, collectively contributing to neuroinflammation, myelin debris accumulation, remyelination failure, and progressive CNS damage.

EV-miR-155-5p was the most consistently and significantly downregulated target, reaching FDR-corrected significance in all three patient comparisons: SPMS vs. HC (FC = 0.35, FDR q = 0.033), RRMS vs. HC (FC = 0.28, FDR q = 0.046), and combined MS vs. HC (FC = 0.31, FDR q = 0.033). *miR-155* is one of the most extensively characterised miRNAs in the context of neuroinflammation, with established roles in destabilising endothelial tight junction proteins and thereby contributing to BBB permeability under inflammatory conditions [20], as well as in promoting a pro-inflammatory myeloid cell phenotype and regulating Th1/Th17 T lymphocyte differentiation (Figure 10) [21,22,23]. Upregulation of *miR-155* in circulating myeloid cells and within active MS lesions has been repeatedly documented [23,24], and cellular *miR-155* expression has been associated with disability scores and lesion activity in MS patients. Against this background, the reduction in EV-miR-155-5p levels may initially appear counterintuitive. However, the distinction between intracellular miRNA expression and vesicular cargo loading is critical: EV biogenesis involves selective, ceramide- and ALIX-dependent sorting mechanisms that do not simply mirror intracellular miRNA abundance. In progressive MS, a shift from the acute relapse-driven inflammation characteristic of RRMS toward a compartmentalised, smouldering pathological process is well established [1,3]. This transition may substantially alter the composition of EV cargo released into the peripheral circulation, reducing *miR-155-5p* loading into secreted vesicles even while intracellular *miR-155* activity remains elevated within CNS-resident inflammatory cells. This interpretation is further supported by the recent Mosora et al. review, which identified *miR-155* among EV-miRNAs showing the most relevant dysregulation at the RRMS-to-SPMS transition [18]. However, we emphasize that this mechanism is currently a speculative inference rather than a demonstrated biological reality in our cohort. Because our total peripheral EV-enriched fraction profiling cannot isolate cellular-origin kinetics, this interpretation must be treated strictly as a hypothesis-generating foundation. Direct verification through neuroimaging inflammatory correlations, fluid biomarkers of neurodegeneration, or cell-specific sorting pipelines is fundamentally necessary to definitively establish its clinical and biological validity.

Neither *miR-30a-5p* nor *miR-146a-5p* reached statistical significance in the cross-sectional analysis, although both trended toward lower expression in MS patients relative to HCs. For miR-146a, this finding is somewhat inconsistent with prior reports identifying miR-146a-3p as specifically downregulated in SPMS compared to RRMS in plasma-based studies [25]. The divergence likely reflects methodological differences, particularly the EV-specific isolation approach used here, which captures only the vesicle-encapsulated miRNA fraction and may not detect changes occurring in the freely circulating pool. The relatively modest fold-changes for both targets and the limited cohort size further constrain the statistical power available for these comparisons. The involvement of *miR-30a-5p* in T lymphocyte regulation [23] and oligodendrocyte maturation is biologically plausible in the MS context, but the current data do not support its utility as a plasma EV biomarker for distinguishing MS disease states (Figure 10).

A central and novel aspect of this study was the longitudinal assessment of EV-miRNA expression before and after one year of siponimod treatment in 11 paired SPMS patients. Siponimod is a selective S1P1 and S1P5 receptor modulator approved for active SPMS, whose mechanism of action is dual: peripherally, it retains autoreactive lymphocytes in secondary lymphoid organs by functionally antagonising S1P1-mediated lymphocyte egress; centrally, it crosses the BBB and engages glial S1P1 and S1P5 receptors, producing anti-inflammatory effects through NFkB inhibition and Nrf2 activation, as well as direct neuroprotective effects on oligodendrocytes and neurons [4,26,27]. In preclinical models, siponimod has been shown to reduce astrogliosis, microgliosis, and cortical neuronal circuit dysfunction, independent of its peripheral immunomodulatory effects [4]. Despite these well-documented mechanisms, no statistically significant change in any of the four EV-miRNAs was detected after one year of treatment (Wilcoxon signed-rank test: *p* ≥ 0.577 for all targets). Several interpretations are possible. First, the EV-miRNA profile may represent a stable molecular correlate of the chronic progressive disease state, less susceptible to short-term modulation by pharmacological intervention than, for example, lesion activity or disability progression measured in clinical trials. Second, the n = 11 paired sample size provides limited statistical power for detecting small treatment-associated changes, particularly given the high intra-individual variability documented for the low-abundance targets *miR-223-5p* and *miR-155-5p*. Third, the absence of a treatment-naïve longitudinal arm means that natural temporal fluctuations in EV-miRNA levels cannot be distinguished from a true pharmacodynamic signal. It is worth noting that miRNA-based pharmacodynamic markers have been identified for other S1P-targeting therapies: miRNA-548a-3p was proposed as a predictor of no evidence of disease activity under fingolimod [28], suggesting that S1P modulators can, under certain conditions, reshape circulating miRNA landscapes. Whether a longer treatment period, a larger cohort, or more sensitive quantification methods such as digital droplet PCR would reveal a siponimod-associated EV-miRNA signature remains an open question for future investigation. Our protocol lacked an untreated SPMS longitudinal arm, an alternative treatment comparator group, and direct correlations with siponimod exposure, peripheral lymphocyte reductions, and objective neurodegenerative markers (such as EDSS progression or MRI lesion tracking), so we cannot establish whether these profiles represent true treatment-insensitive disease-state correlates. The correct conclusion is strictly restricted to the statement that no detectable within-subject change was observed in this specific pilot cohort.

The multivariate analysis provided additional context: PCA of subject-level ΔCt profiles across the four miRNAs explained 62.0% of variance on PC1 and 19.0% on PC2, with SPMS patients showing a partial tendency toward separation along PC1 from HCs, consistent with their globally lower EV-miRNA expression. The absence of a clear three-group separation in PCA space underscores the substantial inter-individual variability in plasma EV-miRNA content, a recognised challenge in the field that is attributable to biological heterogeneity, variability in EV isolation yield, and the near-detection-limit amplification of several targets, particularly *miR-223-5p* [29]. This variability does not invalidate the group-level statistical findings but highlights the need for larger cohort sizes and standardised pre-analytical protocols to establish individual-level diagnostic thresholds.

Our study possesses several key strengths, including its prospective sample collection, longitudinal follow-up, focus on the SPMS population, and investigation of EV-miRNAs in SPMS cases undergoing siponimod therapy, an area for which published data are currently lacking. Collectively, the clinical and biological value of this work is threefold. First, it serves as a crucial hypothesis-generating foundation for progressive MS subtypes; by documenting a statistically significant cross-sectional downregulation of EV-miR-223-5p and EV-miR-155-5p in SPMS patients versus healthy controls (even after strict FDR correction), we provide preliminary peripheral evidence that likely reflects the chronic, compartmentalized neuroinflammatory state characteristic of progressive disease. Second, our findings characterize the molecular stability of these biomarkers, as the stable microRNA profiles observed over a twelve-month therapeutic window—showing no rapid, short-term pharmacodynamic fluctuations—suggest they may serve as indicators of an enduring, baseline disease state rather than volatile markers highly sensitive to brief intervention. Third, this pilot work establishes a rigorous methodological foundation by adhering strictly to the updated MISEV2023 guidelines for EV isolation and physiochemical characterization, providing a reproducible baseline protocol specifically tailored for progressive cohorts under S1P receptor modulation. To our knowledge, this is the first prospective pilot biomarker study that investigates longitudinal EV-miRNAs in SPMS cases undergoing siponimod therapy, providing novel data in an area where published evidence remains scarce.

The interpretation of these findings must be considered alongside several limitations. The cohort sizes are modest, particularly the RRMS comparator group (n = 7) and the paired longitudinal sub-cohort (n = 11), which constrains statistical power and the scope of conclusions that can be drawn. Normalization relied on a single endogenous reference gene (*miR-16-5p*), whose Ct values showed inter-group differences driven by the small RRMS subgroup, a limitation that multi-reference normalization strategies or synthetic spike-in controls should address in future work. The polymer precipitation-based EV isolation method, while practical and scalable, does not discriminate between distinct vesicle subpopulations. Because our protocol evaluates total circulating plasma cargo without localizing cellular tissue of origin or capturing downstream functional consequences, this potential link remains an associative hypothesis. Confirming this relationship requires future validation through matching CSF sampling, the enrichment of CNS-specific EV subpopulations, or targeted functional assays. Finally, the absence of paired clinical and paraclinical data, including EDSS scores, detailed disease duration, MRI lesion burden, smoking status, or absolute lymphocyte count or neurofilament light chain levels, means that the relationship between EV-miRNA profiles and individual disease trajectory remains unexplored. This represents perhaps the most clinically relevant gap in the present work, and prospective studies incorporating structured, protocol-defined collection of these variables alongside EV-miRNA profiling, ideally in larger cohorts, will be needed to determine whether these signatures reflect individual disease trajectories rather than distinguishing patient groups only at the cohort level.

## 5. Conclusions

In conclusion, this small pilot study offers highly preliminary insights into EV-miRNA dynamics within a modest cohort of SPMS patients undergoing siponimod therapy. While our observation of downregulated plasma EV-miR-223-5p and EV-miR-155-5p in SPMS relative to HCs appears to align with their described roles in immune regulation, these exploratory data must be interpreted cautiously, serving only as a tentative basis for their potential association with progressive neuroinflammation. Similarly, the observed stability of these profiles over one year of treatment is strictly preliminary, perhaps hinting at an enduring baseline molecular state rather than a rapid, short-term pharmacodynamic response, though this requires validation in a much larger population. Ultimately, given our limited sample size, these findings do not support definitive clinical conclusions and are presented solely as a hypothesis-generating foundation. They offer a modest, incremental step in expanding the literature, highlighting a clear need for larger, prospective validation studies to determine whether these EV-miRNAs have true clinical utility in monitoring SPMS.

## Figures and Tables

**Table 1:** Descriptive statistics of EV-miRNA expression after *miR-16-5p* normalization.

miRNA	Group	n	ΔCt (Mean ± SD)	ΔCt (Median)	Fold-Change vs. HC (95% CI)
***miR-223-5p***	HC	13	7.12 ± 1.78	7.34	1.00 (reference)
	SPMS	16	9.09 ± 1.90	9.19	0.26
	RRMS	7	8.01 ± 1.22	7.79	0.54
***miR-155-5p***	HC	13	4.21 ± 1.02	4.31	1.00 (reference)
	SPMS	16	5.72 ± 2.00	5.32	0.35
	RRMS	7	6.04 ± 1.82	5.62	0.28
***miR-30a-5p***	HC	13	4.29 ± 1.29	3.97	1.00 (reference)
	SPMS	16	4.71 ± 1.58	4.54	0.74
	RRMS	7	5.34 ± 1.33	4.97	0.48
***miR-146a-5p***	HC	13	3.50 ± 0.71	3.46	1.00 (reference)
	SPMS	16	4.06 ± 1.06	3.94	0.68
	RRMS	7	3.49 ± 0.68	3.51	1.01

HC = Healthy Control; SPMS = Secondary Progressive MS; RRMS = Relapsing–Remitting MS. ΔCt = Ct(target) − Ct(*miR-16-5p*); lower ΔCt = higher relative expression. Fold-change = 2^−ΔΔCt^ relative to HC mean; values < 1 indicate downregulation.

**Table 2:** Omnibus Kruskal–Wallis tests across HC, SPMS, and RRMS cohorts on subject-level ΔCt (*miR-16-5p*-normalized).

miRNA	Kruskal–Wallis H	p-Value	FDR q-Value	Significance
***miR-223-5p***	8.85	0.012	0.024	Significant *
***miR-155-5p***	9.51	0.009	0.024	Significant *
***miR-30a-5p***	3.46	0.177	0.177	n.s.
***miR-146a-5p***	3.58	0.167	0.177	n.s.

* FDR q < 0.05. Benjamini–Hochberg correction applied across the four targets. n = 13 HCs, 16 SPMS patients, 7 RRMS patients. n.s.—not significant.

**Table 3:** Pairwise Mann–Whitney U comparisons versus HC after *miR-16-5p* normalization, with FDR correction.

miRNA	Comparison	FC	p (MW)	FDR q-Value	Significance
***miR-223-5p***	SPMS vs. HC	0.26	0.013	0.026	Significant *
	RRMS vs. HC	0.54	0.351	0.401	n.s.
	SPMS + RRMS vs. HC	0.30	0.012	0.046	Significant *
***miR-155-5p***	SPMS vs. HC	0.32	0.006	0.033	Significant *
	RRMS vs. HC	0.28	0.014	0.046	Significant *
	SPMS + RRMS vs. HC	0.31	0.002	0.033	Significant *
***miR-30a-5p***	SPMS vs. HC	0.74	0.283	0.348	n.s.
	RRMS vs. HC	0.48	0.115	0.229	n.s.
	SPMS + RRMS vs. HC	0.65	0.138	0.246	n.s.
***miR-146a-5p***	SPMS vs. HC	0.72	0.083	0.203	n.s.
	RRMS vs. HC	1.01	0.817	0.871	n.s.
	SPMS + RRMS vs. HC	0.80	0.157	0.251	n.s.

* FDR q < 0.05. FC = fold-change 2^−ΔΔCt^ relative to HC; values < 1 = downregulation. MW = Mann–Whitney. FDR correction applied across all pairwise comparisons. n.s.—not significant.

## Data Availability

The datasets generated and analysed during the current study are available from the corresponding author upon reasonable request.
